# Effects of Pain Reduction by Self-Natural Posture Exercise on Affective Complexity in Women: The Moderating Effect of Self-Regulation

**DOI:** 10.3389/fpsyg.2020.01317

**Published:** 2020-06-26

**Authors:** Jungki Choi, Jiyoo Yoon, Myoungjin Shin

**Affiliations:** ^1^Pacific InterContinental College, Manila, Philippines; ^2^Kookmin University, Seoul, South Korea

**Keywords:** chronic pain, affect, exercise therapy, women, SNPE

## Abstract

This study aimed to investigate the effects of pain reduction and self-regulation efficacy on affective complexity in female patients with chronic pain after participation in an exercise therapy program—Self-Natural Posture Exercise (SNPE)—within the theoretical framework of the Dynamic Model of Affect. A 12-week SNPE program (thrice a week, 70 min per session) was conducted with 101 women with chronic pain lasting longer than 6 months. Pre- versus post-SNPE difference in the correlation between positive affect (PA) and negative affect (NA) was examined through Fisher’s *z* test, and the moderation effect was confirmed through hierarchical regression analysis. Upon completion of the program, participants experienced pain [*M*_pre_ = 5.68 (*SD*_pre_ = 1.96) vs. *M*_post_ = 3.12 (*SD*_post_ = 2.16)] and stress reduction [*M*_pre_ = 2.92 (*SD*_pre_ = 0.95) vs. *M*_post_ = 2.62 (*SD*_post_ = 0.86)], higher satisfaction with life [*M*_pre_ = 4.25 (*SD*_pre_ = 1.20) vs. *M*_post_ = 4.80 (*SD*_post_ = 1.15)], and decreases in the negative correlation between PA and NA (*r*_pre_ = −0.541 vs. *r*_post_ = −0.379). Furthermore, participation in the SNPE program neutralized the impact of PA_post_ on NA_post_ (β = −0.03) in participants with high self-regulation and pain reduction. These results suggest that self-regulation helps to increase SNPE adherence, which would induce pain reduction and restore affective complexity. Based on the strength model of self-control, to increase the pain reduction through exercise therapy, the instructor should ensure that the participants are not being ego depleted.

## Introduction

### The Dynamic Model of Affect and Pain

“Affective complexity” is the key concept underlying the Dynamic Model of Affect (DMA) (Zautra et al., 2002). Complexity comprises differentiation and integration processes; differentiation refers to types and amounts of information discernible in a given situation, while integration refers to the ability to combine different information (Porter and Suedfeld, 1981). From this, it follows that affective complexity means the extent to which differentiation is exercised with regard to positive affect (PA) and negative affect (NA), along with simultaneous integration of many different emotions (Ong et al., 2004). According to Qian et al. (2014), the relationship between PA and NA is independent at high affective complexity, while at low affective complexity, there exists a negative correlation between PA and NA. Zautra et al. (2002) proposed DMA, which focuses on contextual factors (e.g., stressful experiences). In a stress-free situation, high affective complexity takes hold, enabling people to process both PA and NA, without PA and NA interfering with each other (Davis et al., 2004; Qian et al., 2014). However, a stressful situation induces low affective complexity, undermining the ability to process information (e.g., the ability to practice differentiation and integration), which results in a high negative correlation between PA and NA.

Pain is a strong stressor for patients with chronic pain, and it has been studied using DMA. Zautra et al. (2001) measured pain, PA, and NA in patients with rheumatoid arthritis (RA) or osteoarthritis (OA) (Study 1). As a result, an inverse correlation between PA and NA was observed to increase along with a progressive increase in pain intensity every week. In Study 2, they extended their research using a daily measurement of pain, PA, and NA in patients with fibromyalgia (FM) and found that an increase in pain intensity entailed an increase in the inverse correlation between PA and NA (Zautra et al., 2001). Finan et al. (2009) investigated the correlation between PA and NA according to three types of pain (group 1 = OA, group 2 = FM, group 3 = OA/FM) in female patients and found that the tendency of an inverse correlation between PA and NA was more markedly demonstrated in the FM and OA/FM groups, compared to the OA group. This finding may be explained by the fact that pain associated with FM is less predictable compared to OA, with its pain-inducing mechanisms remaining unclear. Therefore, patients with FM are exposed to more high-stress situations (Davis et al., 2004). In a review of research on pain and DMA, Ong et al. (2017) emphasized that chronic pain exists in an informative context, in which affective dynamics predicted by DMA can be observed, as pain is rarely stable and has an unpredictable range of intensity and duration. Recent research (Ciere et al., 2019) has also shown partial support for DMA in the context of chronic migraines.

### Self-Natural Posture Exercise and Pain Reduction

Exercise therapy is less painful than other pain treatment modalities (e.g., pharmacological therapy) and minimizes physical side effects that may be associated with the intervention, as demonstrated by a number of studies (Haahr et al., 2005; Landmark et al., 2011; Daenen et al., 2015). Results of randomized controlled trials conducted to date have demonstrated physical exercise as a viable therapeutic modality with beneficial effects on chronic pain, physical function, sleep quality, and cognitive function (Hagen et al., 2012; Kayo et al., 2012). The meta-regressions indicated that increases in knee extensor strength would be necessary for a likely concomitant beneficial effect on pain (Bartholdy et al., 2017). An important element for enhancing the effectiveness of exercise therapy is designing a program with moderate intensity (avoiding high intensity) (Jones et al., 2006; Nelson et al., 2007) and prioritizes flexibility training (Gavi et al., 2014). Flexibility training includes exercise to improve the range of movement in the joint and to reduce muscle stiffness (Ambrose and Golightly, 2015).

SNPE (Yoon et al., 2019) is a series of exercise programs inspired by orthodontic principles.^1^ It is performed with belts worn around one’s hips (pelvic correction belt) and legs (right posture belt). Other tools, such as a danason (a neck massage tool) and wave pillow, are used for posture self-correction, loosening up tense muscles around the spine, and gradually correcting spinal deformity. A typical SNPE program is composed of eight exercise units including SNPE movement 1, SNPE movement 2, SNPE movement 3, and SNPE movement 4, cervical movement (C-move), thoracic movement (T-move), lumbar movement (L-move), and sacrum–coccygeal movement (SC-move) (Yoon et al., 2019). SNPE is an exercise therapy program optimized for pain reduction that can be practiced anywhere, when equipped with portable belts and other tools. SNPE is a self-directed workout, a low-cost solution to posture correction and pain management, and low- to moderate-intensity flexibility-based muscle strength training. Thus, SNPE can be considered as one exercise therapy modality ideal for chronic pain management and pain reduction, which was demonstrated through empirical studies (Yoon et al., 2019).

### Self-Regulation and Exercise Participation

Self-regulation is defined as an ability to regulate or inhibit impulsive drives to obtain desired results or accomplish envisioned goals (Muraven and Slessareva, 2003; Baumeister and Vohs, 2007). Self-regulatory failure is closely associated with problems and difficulties people encounter in their lives such as excessive debt, substance abuse, obesity, unplanned pregnancy, sexually transmitted infections, crime, and violence (Muraven and Baumeister, 2000). High self-regulation competence leads to behaviors contributing to a successful and healthy life such as academic and occupational accomplishment, smooth interpersonal relationships, and high psychological well-being (Duckworth and Seligman, 2005; Hofmann et al., 2014).

According to social cognitive theory, people’s behavior is controlled by volitional beliefs, motives, intentions, and expectations (Ajzen, 1991). This approach implies that behaviors such as exercise participation require considerable planning and deliberation prior to initiation (Hagger et al., 2002; Chatzisarantis and Hagger, 2009). Empirical studies applying the social cognitive model also verified that self-regulation was closely associated with exercise participation and intent to continue (Ahn et al., 2016; Hallion et al., 2019). A study by Stadler et al. (2009), applied with a longitudinal research design, showed that female participants in the self-regulated manipulation group performed an hour more of physical activity per week than the control group. In addition, in studies conducted with participants with cancer (Ungar et al., 2015) and multiple sclerosis (Cederberg et al., 2018), self-regulation was found to be an effective intervention strategy that enhances physical activity and exercise participation. Therefore, self-regulation is an important psychological variable that has a positive effect on exercise participation and continuance.

### Limitations of Previous Research and Purpose of This Study

Research on affective complexity conducted to date has examined, within the framework of the DMA, emotional processing in various stress situations, adopting two research approaches. The first approach has been to study the relationships between NA and PA in various environmental contexts (e.g., stress situations) (Potter et al., 2000; Zautra et al., 2001). The second approach has investigated the effects of stress-coping resources on affective complexity. Several stress-coping strategies (e.g., sports with friends and shopping) were found to help restore affective complexity (Zautra et al., 2005, 2007; Qian et al., 2014). Most participants in DMA-related studies were patients with chronic pain, and one of the most serious stressors for them was persistent pain. However, previous studies failed to examine the effects of recovery of affective complexity by directly inducing reduction in pain, which was the key stressor in patients with chronic pain.

In summary, the purpose of this study was to find answers to two research questions such as would people with chronic pain be able to process various emotional information in complex situations as efficiently as people without chronic pain? What may be the psychological factors that can help them perform efficient information processing and decision-making in complex situations? Prompted by these questions, the present study was conducted on women with chronic pain to investigate the effects of pain reduction on their effective complexity, based on the DMA. Additionally, the present study investigated the effects of self-regulation associated with participation in exercise therapy in the relationship between PA and NA. The following hypotheses can be formulated: (1) participants with chronic pain will have fewer negative emotions upon completion of the SNPE program; (2) participants with chronic pain will have reduced pain upon completion of the SNPE program; (3) the strength of the negative correlation between PA and NA at baseline will become weak upon completion of the SNPE program; and (4) the higher the self-regulation and the greater the pain reduction upon completion of the SNPE program, the weaker the effect of PA on NA.

## Materials and Methods

### Participants

The study was conducted with 107 out of the 125 women who were enrolled in the SNPE Training Center, excluding 18 participants who had experienced pain for <6 months. Thus, in this study, there were 107 participants who had lasting pain (≥6 months and greater than or equal to moderate pain level in the lower back, upper body (shoulder, neck, arms), or lower body (knees, thighs, hips)]. According to Boonstra et al. (2014), moderate pain was selected when the Visual Analogue Scale (VAS) score was ≥4. After excluding six participants who did not complete the training for personal reasons (e.g., pregnancy, travel abroad, and business trips), data from 101 participants (lower back, *n* = 25; upper body, *n* = 58; lower body, *n* = 18) were used in the final analysis. A G^∗^Power analysis revealed that our sample size was sufficient to reveal at least a moderate effect in the present study (Davis et al., 2004; parameters: *f* = 0.23, α = 0.05, 1 −β = 0.80, number of predictors = 12; exact sample advised: *N* = 85). Participants’ mean pain duration was 75.08 months (*SD* = 75.82), and participants’ mean age was 33.06 years (*SD* = 8.64). The study was conducted with participants who voluntarily consented to participate after being given detailed information on the study’s purpose and process. This study was approved by the research ethics committee from Seoul National University. All participants gave written informed consent to participate.

### Instruments

#### Perceived Pain

Participants’ perceived pain was measured using the VAS developed by Huskisson (1974). The VAS, which measures the intensity of a person’s self-perceived pain, is an 11-point scale ranging from 0 to 10, with 0 being no pain and 10 being the most intense pain imaginable. If there were participants with pain in several areas, it was requested that participants select only one area with the highest pain intensity, and its pain level was measured. The VAS score according to the pain location of the participants [*M*_lower back_ = 5.04 (*SD*_lower back_ = 1.79), *M*_upper body_ = 5.97 (*SD*_upper body_ = 2.02), *M*_lower body_ = 5.61 (*SD*_lower body_ = 1.88)] was more than 5 points, which was on the moderate pain level.

#### PA and NA

Participants’ PA and NA were measure using the Korean version of the Depression Adjective Checklist with 32 descriptors (Lee, 1999): 21 descriptors for the state of NA (e.g., exhausted, depressive, stuck, etc.) and 11 descriptors for the state of PA (e.g., warm, light, proud, etc.). In this study, each descriptor was given 1 point. Considering 1 point assigned to each descriptor, the highest scores for NA and PA were 21 and 11 points, respectively.

#### Self-Efficacy to Regulate SNPE

Self-regulation ability during the 12-week SNPE program was measured using the questionnaire modified from the Self-Efficacy to Regulate Exercise (SRE) developed by Bandura (2006), to suit the purpose of our study. The questionnaire consists of 18 items answers using a scale ranging from 0 to 100%, rating the degree to which participants believed they could participate in the SNPE program even under adverse circumstances. Reliability of the SRE SNPE Questionnaire was α_pre_ = 0.895 at baseline and α_post_ = 0.801 upon completion of the SNPE program.

#### Satisfaction With Quality of Life

Participants’ level of satisfaction with their quality of life was measured using the Korean version of the Satisfaction with Life Scale (SWLS) developed by Diener et al. (1985). The SWLS is a short instrument consisting of five items designed to measure the degree of overall satisfaction with one’s quality of life: (1) in most ways, my life is close to ideal; (2) the conditions of my life are excellent; (3) I am satisfied with my life; (4) so far, I have gotten the important things I want in life; and (5) If I could live my life over, I would change almost nothing. Responses are rated using a 7-point Likert scale. Reliability of the SWLS was α_pre_ = 0.886 at baseline and α_post_ = 0.905 upon completion of the SNPE program.

#### Perceived Stress

To measure participants’ self-rated stress levels, we used the Korean version of the Perceived Stress Scale (K-PSS), based on the original scale developed by Cohen and Williamson (1988). The K-PSS comprises five NA-related items: (1) In the last month, how often have you been upset because of something that happened unexpectedly; (2) in the last month, how often have you felt that you were unable to control the important things in your life; (3) in the last month, how often have you felt nervous and “stressed;” (4) in the last month, how often have you felt confident in your ability to handle your personal problems; and (5) in the last month, how often have you coped efficiently with important changes in your life? The K-PSS was measured based on answers rated using a 5-point scale (1 = *never*; 5 = *very often*). Reliability of the K-PSS was α_pre_ = 0.851 at baseline and α_post_ = 0.889 upon completion of the SNPE program.

### Research Design and Procedure

#### Research Design

This study used repeated measures design to examine the change in correlation between PA and NA in participants with chronic pain after SNPE. Repeated measures design involves multiple measures of the same variable taken on the same or matched subjects either over two or more time periods and is collected by a longitudinal study approach in which change over time is assessed. This type of design is often regarded as superior to a cross-sectional design because it enables processes and causes of change within individuals to be identified (Deschenes, 1990). In this study, there was no control group to contrast the effectiveness of the treatment. One of the reasons is that assigning participants suffering from chronic pain as a control group (e.g., no treatment) for research could be a violation of research ethics.

#### Procedure

The SNPE program presented in Table 1 was administered to participants three times a week, for 70 min per session, over a period of 12 weeks. Prior to the first session, we measured participants’ baseline levels of perceived pain, affect, self-efficacy to regulate SNPE, life satisfaction, and stress. The first session is an orientation, focusing on instructor introduction, class execution methods, and other notices, and the program of Table 1 starts from the second session. Baseline measurements were conducted during the first session. Upon completion of the 12-week program, postintervention measurements were performed using the same items. We recruited four certified SNPE instructors. In conducting a preliminary meeting with four certified SNPE instructor, the SNPE training program in Table 1 was applied equally to all participants. In addition, participants were asked to write an exercise log, and the contents were checked to make sure the difference in the amount of exercise and intensity between participants was minimized. The four SNPE instructors were not given information on the detailed research objectives and process to prevent measurement bias (e.g., from words or actions that may taciturnly provide feedback to participants or induce feedback from them). In order to reduce measurement bias based on teacher–student rapport formed during the training period, or by virtue of the authority of the instructor, the assistant researcher visited the training sites and conducted questionnaire-based measurements. The last four digits of participants’ cell phone numbers were used as their respective identities.

**TABLE 1:** The SNPE program.

Category	Type of exercise	Frequency/duration
Warm-up (5 min)	Muscular fascia relaxing stretch using the SNPE wave pillow.	5 min
Main exercise (50 min)	SNPE movement 4: SNPE Spinal rock with SNPE body correction belts.	100–200 times
	SNPE movement 1: Invisible chair pose with both hands clasped behind the back.	30 s × 5 set
	SNPE movement 3: Reverse leg raise with bent knee, using SNPE belts.	30 s × 5 set
	SNPE L-move: Placing the SNPE wave pillow under the posterior superior iliac spine horizontally, moving legs up and down, to restore the original curve of the back.	50–100 times
	SNPE T-move: Placing the SNPE wave pillow under the back vertically to loosen up tight back muscles.	R) 100 times L) 100 times
	SNPE movement 2: Lying back while kneeling, thighs tied with SNPE belts.	1–3 min
Cool down (15 min)	SNPE C-move: Placing the SNPE wave pillow or Danason under the neck. Moving the head from left to right to gently loosen muscles around the neck. SNPE SC-move: Placing the SNPE wave pillow under the pelvis to loosen up the muscles around the sacrum and coccyx.	10 min
	Rest	5 min

*SNPE, self-natural posture exercise; R, right; L, left; L-move, lumbar movement; T-move, thoracic movement; C-move, cervical movement; SC-move, sacrum-coccygeal movement.*

### Data Analysis

Basic statistical analysis, correlation analysis, and reliability analysis were carried out using SPSS 18.0 software, and statistical significance of the differences between correlation coefficients was tested with Fisher’s *z* test. A paired *t* test was used to compare the mean of pain, life satisfaction, stress, and affect from related sample. The effect size was calculated as Cohen’s *d*. The moderated moderation effect was determined using Model 3 of PROCESS developed by Hayes (2017). Since PROCESS does not provide standardized regression coefficients in the regression analysis, we converted input variables into standard scores. The standardized independent variables were introduced into the equation in five successive steps. In the first step (1), covariate variable (CV) such as age, Stress_pre_, SWLS_pre_, PA_pre_, and NA_pre_ were introduced to control their possible influence on dependent variable (DV) (e.g., NA_post_). Next (2), independent variable (IV) such as PA_post_, followed by (3) the primary moderator variable (PMV) (e.g., self-regulation efficacy), followed by (4) the secondary moderator variable (SMV) (e.g., pain reduction), and finally (5), interaction term (IT) such as two-way (PA_post_ × self-regulation efficacy, PA_post_ × pain reduction, self-regulation efficacy × pain reduction) and three-way interaction (PA_post_ × self-regulation efficacy × pain reduction). *Post hoc* regression was used to visually express the significance of the interaction. Specifically, separate lines of regression were generated from the regression equation to represent the PA_post_ and NA_post_ relationship at relatively high (+1 *SD*) and relatively low (-1 *SD*) levels of the moderator variables. The statistical significance level was set at 0.05.

## Results

### Pre- vs. Post-SNPE Differences in Pain, Life Satisfaction, Stress, and Affect

A paired sample *t* test, as shown in Table 2, was conducted to determine changes in pain levels and psychological variables between pre-SNPE (baseline) and post-SNPE (upon completion of the SNPE program) values. As a result, mean VAS score was found to have decreased from 5.68 (*SD* = 1.96) to 3.12 (*SD* = 2.16), with the difference being statistically significant [*t*(100) = 9.67, *p* < 0.001, *d* = 1.24). On the other hand, the SWLS (*M*_pre_ = 4.25, *SD*_pre_ = 1.20 vs. *M*_post_ = 4.80, *SD*_post_ = 1.15, *t*(100) = 5.55, *p* < 0.001, *d* = 0.46) and PA (*M*_pre_ = 4.81, *SD*_pre_ = 3.18 vs. *M*_post_ = 7.29, *SD*_p__ost_ = 2.94, *t*(100) = 7.33, *p* < 0.001, *d* = 0.80) increased with statistical significance. A statistically significant decrease was found in stress (*M*_pre_ = 2.92, *SD*_pre_ = 0.95 vs. *M*_post_ = 2.62, *SD*_post_ = 0.86, *t*(100) = 3.83, *p* < 0.001, *d* = 0.33) and NA (*M*_pre_ = 6.14, *SD*_pre_ = 5.61 vs. *M*_post_ = 4.12, *SD*_post_ = 4.59, *t*(100) = 3.76, *p* < 0.001, *d* = 0.39). However, no statistically significant difference was found in self-regulation efficacy (*M*_pre_ = 65.80, *SD*_pre_ = 19.24 vs. *M*_post_ = 69.42, *SD*_post_ = 20.24, *t*(100) = 1.55, *p* = 0.126, *d* = 0.18). Thus, Hypotheses 1 and 2 were supported, with strong statistical significance.

**TABLE 2:** Paired samples *t*-test for Pre- vs. Post-SNPE participation in pain, life satisfaction, stress, and affect.

Variable	Pre		Post		*t* (*df* = 100)	Cohen’s *d*
		
	*M*	*SD*	*M*	*SD*		
VAS	5.68	1.96	3.12	2.16	9.67***	1.24
SWLS	4.25	1.20	4.80	1.15	5.55***	0.46
PA	4.81	3.18	7.29	2.94	7.33***	0.80
NA	6.14	5.61	4.12	4.59	3.76***	0.39
Stress	2.92	0.95	2.62	0.86	3.83***	0.33

VAS, visual analogue scale; SWLS, satisfaction with life scale; PA, positive affect; NA, negative affect. *** *p* < 0.001.

### Pre- vs. Post-SNPE Differences in Correlation Between PA and NA

Pre- vs. post-SNPE differences in correlation between PA and NA were investigated by calculating the Pearson’s correlation coefficients. Pre- and post-SNPE correlations between PA and NA were *r*_pre_ = -0.541 (*p* < 0.001; *R*^2^ = 0.292) and *r*_post_ = −0.379 (*p* < 0.001; *R*^2^ = 0.143), respectively, which suggested that the correlation between PA and NA decreased over the course of the 12-week SNPE program. This pre- vs. post-SNPE difference in correlation coefficients was found to have marginal statistical significance (*z* = 1.45, *p* < 0.10) by Fisher’s *z* test. This implied that the strength of association between PA and NA decreased from moderate (*r*_pre_ = -0.541) to weak (*r*_post_ = -0.379) through participation in the SNPE program, thus supporting Hypothesis 3.

### Moderated Moderation Effect

We tested the moderated moderation effect by which the conditional interaction effect between PA and self-regulation efficacy (PMV) on NA varied depending on the level of pain reduction^2^ (SMV). As a result, as shown in Table 3, the total variance of the dependent variable NA_post_ (post-SNPE NA) was explained by the input variables including CV, IV, PMV, and SMV (explanatory power = 47%; *R*^2^ = 0.47), with statistical significance [*F*(12, 88) = 6.38, *p* < 0.001). Although PA_post_ had a statistically negative effect on NA_post_ (β = -0.34, *t* = 3.71, *p* < 0.001), the effects of self-regulation efficacy_pre_ (β = -0.07, *t* = 0.78, *p* = 0.432) and pain reduction (β = -0.04, *t* = 0.42, *p* = 0.676) on NA_post_ did not reach statistical significance. The slopes of the three-way interaction variable (β = -0.18, *t* = 2.39, *p* < 0.05) and Δ*R*^2^ [*F*(1, 88) = 5.73, *p* < 0.05] were all statistically significant, thus verifying the existence of the moderation effect.

**TABLE 3:** Results from multiple regression analysis examining the moderated moderation effect of PA_post_ on NA_post_ by self-regulation efficacy and pain reduction.

	Dependent variable	NA_post_	
		
		β	*R*^2^ (Δ*R*^2^ for a × b × c)
CV	Age	0.02	0.47*** (0.03*)
	Stress_pre_	0.15	
	SWLS_pre_	0.02	
	PA_pre_	0.36**	
	NA_pre_	0.58***	
IV	PA_post_^a^	−0.35***	
PMV	Self-Regulation Efficacy_pre_^b^	–0.07	
SMV	Pain reduction^c^	0.04	
IT	a × b	0.022	
	a × c	–0.12	
	b × c	0.13	
	a × b × c	−0.18*	

CV, covariate variable; IV, independent variable; PMV, primary moderator variable; SMV, secondary moderator variable; IT, interaction term; SWLS, satisfaction with life scale. * *p* < 0.05, ** *p* < 0.01, *** *p* < 0.001.

A *post hoc* simple regression analysis was performed to interpret the moderation effect. First, analysis of the interaction effect between self-regulation efficacy and PA_post_ on NA_post_ revealed that PA_post_ had a negative effect on NA_post_ (β = -0.34) in both participants with low self-regulation efficacy (mean self-regulation efficacy - 1SD) and high self-regulation efficacy (mean self-regulation efficacy + 1SD). In contrast, the conditional effect of the interaction between self-regulation efficacy and PA_post_ on NA_post_ varied depending on pain reduction. In patients with high pain reduction and self-regulation, the slope (β) of PA_post_ over NA_post_ was −0.03 (*t* = 0.16, *p* = 0.874), which was below the significance cutoff, thus supporting Hypothesis 4. In contrast, in patients with a high degree of pain reduction and low self-regulation efficacy, the slope (β) of PA_post_ over NA_post_ was −0.43 (*t* = 2.65, *p* < 0.01), with statistical significance. Among participants whose post-SNPE pain intensity increased relative to pre-SNPE pain intensity, PA_post_ had a negative effect on NA_post_ in both those with low self-regulation efficacy (β = −0.31, *t* = 2.05, *p* < 0.05) and those with high self-regulation efficacy (β = −0.62, *t* = 3.80, *p* < 0.001).

## Discussion

This study examined the benefits of SNPE participation in women suffering from chronic pain as well as its impact on the recovery of affective complexity. After SNPE participation, participants’ pain and stress were reduced, satisfaction with life was increased, and affective complexity was restored. In particular, participants with high pain reduction due to SNPE participation and high self-regulation experienced enhanced recovery of affective complexity. A detailed discussion on the results of this study is as follows.

### SNPE Participation and Pain Reduction

The results of the present study demonstrated the beneficial effects of SNPE as exercise therapy in relieving chronic pain, thus supporting the findings of previous studies indicating that exercise therapy has a positive effect on pain reduction (Haahr et al., 2005; Landmark et al., 2011; Tekur et al., 2012; Daenen et al., 2015; Ekici et al., 2017). According to the findings of previous studies, in order for exercise therapy modalities to be effective in relieving pain, flexibility training should be prioritized over muscle strength training (Gavi et al., 2014) and low-to-moderate intensity exercises over high-intensity exercises (Jones et al., 2006; Nelson et al., 2007). SNPE satisfies both conditions, and its pain reduction efficacy was demonstrated in a study conducted by Yoon et al. (2019). Considering the results of our study and the prerequisites of exercise therapy for pain reduction as presented in previous studies, SNPE can be considered an exercise therapy that helps relieve pain.

SNPE tools including a danason, a wave pillow, a belt, and a band are also expected to help reduce pain. Danasons and wave pillows are designed with solid materials to relax tight muscles, and belts and bands correct skewed postures such as with the spine and pelvis. Therefore, it can be seen that the use of tools to help muscle relaxation and posture correction can be considered to help reduce pain by forming a natural posture.

### Relationship Between Chronic Pain and Uncertainty Within the Scope of DMA

Participation in the SNPE program administered in our study resulted in decreased pain, stress, and NA and increased life satisfaction and PA. These results can be viewed as a phenomenon, manifested as a result of resolving stress and uncertainty, as presented in DMA. People living with chronic pain are exposed to a high-uncertainty situation of not knowing how long the pain will last. DMA proposes the need for rapid information processing to be prioritized over differentiated evaluation of stimuli under conditions of stress and uncertainty (Davis et al., 2004). This suggests that, in a situation characterized by high stress and uncertainty, individuals need to cope with NA more strongly by focusing more on potential threats to their well-being instead of expending their resources for complex and time-consuming processing (e.g., simultaneously conducting differentiated evaluation of multiple stimuli) (Davis et al., 2004). In this context, participants’ low life satisfaction and high uncertainty regarding daily stress and pain before SNPE participation can be interpreted to have induced them to process NA-related information more rapidly, leading to higher NA and lower PA. Furthermore, the post-SNPE decrease in NA and increase in PA can be explained by higher life satisfaction and lower levels of uncertainty, stress, and pain as a result of the SNPE training.

### Effects of Self-Regulation and Pain Reduction on Affective Complexity

In DMA, stressors narrow people’s attention and compel them to focus on an immediate need to cope with the stressor through preferentially processing NA, at the expense of PA, resulting in high-level NA accompanied by low-level PA (Qian et al., 2014; Ong et al., 2017). DMA-based previous studies (Finan et al., 2009; Qian et al., 2014) adopted a cross-sectional approach of using a regression analysis model, which assumed the causal effect of PA (independent variable) on NA (dependent variable) and investigating the process of affective complexity and affective collapse. In the present study as well, the regression model for the effects of PA_post_ on NA_post_ was used to test differences depending on self-regulation and pain reduction.

The results of our study revealed that an inverse correlation between PA and NA still remained upon completion of the SNPE program, although the level of inverse correlation was reduced to a certain degree, compared to baseline levels. Table 3 shows the presence of negative impact of PA_post_ on NA_post_, which allows for the assumption that participation in the 12-week SNPE program contributed to pain reduction, but not to full restoration of affective complexity. However, Table 3 also shows a moderation effect, the interpretation of which led us to a new discovery. In a *post hoc* simple regression analysis, among the participants who achieved a high level of pain reduction, the impact of PA_post_ on NA_post_ was not statistically significant when self-regulation ability was high, but significant when self-regulation ability was low. This may be explained by another mechanism contributing to the formation of affective complexity, rather than the effect of self-regulation on higher participation compliance. One possible answer is the association between self-regulation and individual participants’ cognitive processing styles.

Davis et al. (2004) noted that cognitive processing style plays an important role in the inverse correlation between PA and NA and that the correlation between PA and NA converges toward zero as cognitive processing simplifies. In the social cognitive model, individuals with high self-regulation are characterized by considerable planning and deliberation regarding goals and outcomes of envisioned actions (e.g., exercise participation) before initiation (Hagger et al., 2002; Chatzisarantis and Hagger, 2009). This allows for the assumption that participants with high self-regulation had a relatively simple cognitive processing style. Therefore, in spite of pain reduction through regular SNPE participation, there is a negative relationship between PA and NA because the cognitive processing process is not simplified when the participants’ self-regulation is low. In order to restore the affective complexity of chronic pain participants through exercise therapy, it may be effective to apply psychological strategies (e.g., goal setting) to help simplify cognitive processing, and not just to induce pain reduction.

### Strengths and Implications

Among DMA-related studies conducted thus far, few have explained the phenomenon induced by reducing chronic pain within the theoretical scope of DMA. A number of studies investigated the impact of stress-coping resources on affective complexity in patients with chronic pain when facing everyday stressors (Zautra et al., 2005, 2007). Our study differentiates itself from these studies in that it verified the phenomenon by conducting an empirical–experimental study using actual training sessions of exercise therapy, namely, SNPE, thus retesting the theoretical validity of DMA and adopting a new approach.

Results of this study show that, in order to recover the affective complexity of patients with chronic pain, pain reduction as well as high self-regulation ability to participate in exercise therapy programs are important. These results indicate that it is necessary to consider ways to increase participants’ self-regulation ability when constructing exercise therapy programs for pain reduction. The conditions of exercise therapy for pain reduction presented so far were exercise intensity (i.e., low to moderate intensity) and exercise type (i.e., flexibility training) (Gavi et al., 2014), while the importance of psychological factors such as self-regulation has not been considered. However, the effectiveness of self-regulation strategies (e.g., goal setting, goal pursuit, self-monitoring, etc.) to continue and promote exercise participation has been demonstrated (Ungar et al., 2015). Therefore, the inclusion of self-regulation strategies in the composition of exercise therapy programs will help to restore the affective complexity of patients with chronic pain.

According to the strength model of self-control (Englert, 2016), everybody has self-regulatory resources, and the state of self-regulation resource depletion is ego depletion (Hagger et al., 2010). In other words, inhibition of various instantaneous desires depletes self-regulatory resources, and in a state of ego depletion with all self-regulatory resources exhausted, efficient self-regulatory behavior cannot be performed. Toering and Jordet (2015) found that the more self-regulatory resources individuals possess, the more actively they participate in physical exercise and the less time they spend on social recreational activities. Therefore, those participating in exercise therapy should be careful and not become ego depleted and, if ego depleted, must try to recover the depleted self-regulating resource as soon as possible through sufficient rest (e.g., watching movies, going on a trip, enjoying other leisure activities, etc.). The instructor should ensure that the participants are not being ego depleted, and participants also need to bear in mind that if one is ego depleted, it is difficult to experience pain reduction through exercise therapy.

### Limitations and Future Research Directions

We could not reflect gender difference in determining the relationship between pain reduction and affective complexity. Qian et al. (2014) noted that the stronger the stressor, the greater the decrease in affective complexity in women, compared to men, with women showing higher pain sensitivity (Fillingim et al., 2009). Considering such views of previous researchers, it is inappropriate to expand the results of our study to men with chronic pain. Future studies will need to examine the gender-dependent correlation between pain reduction and affective complexity.

Based on longitudinal data from 12 years (1998–2010; *n* = 19,776), which examined the effects of sociodemographic variables (e.g., gender, education, socio-economic status and ethnicity) on pain (Grol-Prokopczyk, 2017), women and Hispanics suffered more than men and African-Americans, and the higher the level of education and socio-economic status, the lower the pain. In this study, there was a lack of consideration for the relationship between sociodemographic variables and pain. In the follow-up study, it will be necessary to examine the effect of interaction between sociodemographic variables and exercise therapy on pain reduction.

This study was conducted with women suffering from chronic pain of unknown etiology in everyday life for over 6 months, without clinical diagnosis. Participants in previous studies (Zautra et al., 2002; Davis et al., 2004; Finan et al., 2009) were clinically diagnosed patient groups with different types of chronic pain—such as OA, FM, and RA—which allowed for a comparison of the effects of pain reduction on affective complexity in patients with different types of chronic pain. In reality, there are people living with chronic pain without clinical diagnosis. In the light of this, our study investigated DMA theory with participants with undiagnosed chronic pain, which can also be considered to have contributed to expanding research participant population and enhancing external reliability. Nevertheless, future studies should be conducted with clinically diagnosed populations in order to investigate the effects of pain reduction on affective complexity, depending on types of chronic pain, after participation in exercise therapy.

Finally, this study is not a total control-treatment intervention. Therefore, it is not appropriate to understand or interpret the results of this study as a cause–effect relationship. In order to provide strong evidence for the pain reduction effect and the recovery of complexity due to SNPE participation, further studies that include control groups should be conducted.

## Conclusion

This study investigated the effects of pain reduction and self-regulation on affective complexity in female patients with chronic pain after participating in a 12-week SNPE program within the theoretical framework of DMA. Upon completion of this program, participants experienced reduced pain, stress, and increased life satisfaction, as well as a decrease in the negative correlation between PA and NA, which indicated that participation in SNPE training contributed to the formation of high affective complexity. Furthermore, the impact of PA_post_ on NA_post_ no longer existed in participants with high self-regulation regarding participation in SNPE training.

## Data Availability Statement

The original contributions presented in the study are included in the article, further inquiries can be directed to the corresponding author/s.

## Ethics Statement

The studies involving human participants were reviewed and approved by Kookmin University IRB. The patients/participants provided their written informed consent to participate in this study.

## Author Contributions

JC contributed to the literature review and supervision. JY analyzed and interpreted the data. MS wrote the original draft preparation and edited the manuscript. All authors have read and agreed to the published version of the manuscript.

## Conflict of Interest

The authors declare that the research was conducted in the absence of any commercial or financial relationships that could be construed as a potential conflict of interest.

## Footnotes

1 SNPE was developed on the assumption that exercise with belts and other tools (e.g., a danason, a wave pillow, etc.), such as with the use of external force (e.g., springs and wires), could correct misaligned teeth and reduce pain by correcting spinal and pelvic deformations.

2 Since pain reduction was calculated by subtracting VAS_pre_ from VAS_post_, a lower value indicates a higher effect of pain reduction efficacy.
